# Are Drinking Cognitions Associated with Marijuana and Concurrent Alcohol and Marijuana Use among Adolescents and Young Adults?

**DOI:** 10.26828/cannabis/2022.01.006

**Published:** 2022-02-15

**Authors:** Ricarda K. Pritschmann, Nioud Mulugeta Gebru, Dana M. Litt, Zhengyang Zhou, Melissa A. Lewis

**Affiliations:** 1 University of Florida, Department of Health Education & Behavior, Gainesville FL; 2 University of Florida Center for Addiction Research and Education, Gainesville FL; 3 University of Florida Center for Behavioral Economic Health Research, Gainesville FL; 4 University of Florida Clinical and Translational Science Institute, Gainesville FL; 5 University of North Texas Health Science Center, Department of Health Behavior and Health Systems, School of Public Health, Fort Worth, TX; 6 University of North Texas Health Science Center, Department of Biostatistics and Epidemiology, School of Public Health, Fort Worth, TX

**Keywords:** alcohol, marijuana, prototype willingness model, adolescents, young adults

## Abstract

**Background.:**

Concurrent use of alcohol and marijuana (i.e., CAM use) is the most common poly-drug use pattern among adolescents and young adults and is associated with negative outcomes. Research indicates that Prototype Willingness Model (PWM) drinking cognitions are associated with alcohol use. This secondary analysis was conducted to explore cross-sectional associations between PWM drinking cognitions, alcohol, marijuana, and CAM use.

**Methods.:**

Adolescents and young adults between 15-25 years (*N* = 124, *M*_*age*_ = 18.7) completed a baseline assessment as part of a larger study, including questions on alcohol and marijuana use, and PWM drinking cognitions.

**Results.:**

In the social reaction pathway, descriptive norms, perceived vulnerability, and prototype favorability, but not willingness were associated with greater alcohol use, whereas in the reasoned pathway attitudes and intentions were associated with frequency of drinking whereas injunctive norms were not. Both willingness and intention to drink were related to marijuana and CAM use when controlling for alcohol use frequency. Greater willingness to drink was the only significant predictor of marijuana use, and only descriptive norms predicted CAM use. However, of the cognitions within the reasoned pathway, greater attitudes toward drinking and drinking intention were related to greater marijuana and CAM use. Results also indicated that CAM users displayed higher levels of certain risk cognitions than non-users or single substance users.

**Conclusions.:**

Findings support and extend the utility of the PWM by indicating that specific alcohol cognitions are associated with alcohol, marijuana, and CAM use in adolescents and young adults.

## Alcohol, Marijuana and Concurrent Alcohol and Marijuana Use

Alcohol and marijuana are the most commonly used substances among adolescents (Johnston et al., 2020) and young adults (Schulenberg et al., 2020). While adolescent alcohol use has declined over the past decade (Patrick et al., 2017), more than a third (35.9%) of 8th - 12th graders still report ever drinking alcohol (Johnston et al., 2020). Unlike alcohol use, marijuana use has increased in the past decade among adolescents (ages 15 – 18 years) and young adults (ages 18 – 29 years; Johnston et al., 2020, Schulenberg et al., 2020). Indeed, in 2019, one if four (25.2%) of high school students reported using marijuana in the past year (Schulenberg et al., 2020). The past decade has also seen decriminalization and legalization of medicinal and recreational marijuana use across the U.S. (Hall & Lynskey, 2016; Yu et al., 2020), which have been associated increased risk of cannabis use disorder among adolescents, but not in young adults (Cerdá et al., 2017). Alcohol and marijuana use by adolescents and young adults are associated with several short- and long-term consequences including compromised short-term memory and motor coordination, increased likelihood of future dependence, poor educational outcomes, and low life satisfaction (Arria et al., 2013; Suerken et al., 2016; Volkow et al., 2014).

Adolescents and young adults may use only alcohol, only marijuana, or use alcohol and marijuana concurrently. Concurrent use of alcohol and marijuana (i.e., CAM) refers to using marijuana and alcohol within the same period of time (e.g., past month, past year) with non-overlapping effects (Patrick et al., 2018). Data from national surveys indicate that 1.7% and 7.7% of adolescents and young adults, respectively, report CAM use (Patrick et al., 2018; Subbaraman & Kerr, 2015). CAM use is associated with exacerbated negative consequences and health risks compared to alcohol and marijuana use alone. For example, CAM users report more problems, higher alcohol dependency and consequences, and higher sexual risk taking compared to alcohol only users (Cummings, et al., 2019; Saha et al., 2018; Shillington & Clapp, 2001; 2002). In addition, CAM use is related to greater social problems (e.g., fights, and work or relationship problems) than alcohol-only use (Subbaraman & Kerr, 2015). Lastly, substance use outcomes are worse for young adults who are diagnosed with both alcohol and marijuana use disorder (i.e., dual use disorder), and those with dual diagnoses also report greater drinking intensity (i.e., number of drinks consumed per binge episode) than those with a single use disorder (Hayaki et al., 2016). Thus, identifying risk factors for CAM use among adolescents and young adults is of critical importance to improve interventions targeting CAM (Yurasek et al., 2017).

The literature indicates that alcohol and drug use trajectories across adolescence and young adulthood are related (Pape et al., 2009; Schulenberg & Maggs, 2002; Wiesner & Windle, 2004) such that individuals who use alcohol frequently are also more likely to also use other substances at high rates (Barrett et al., 2006; Derefinko et al., 2016). Research suggests that adolescents and young adults are more likely to report being an exclusive user of alcohol or CAM user compared to being an exclusive marijuana user (Cohn et al., 2016; Patrick et al., 2018). Research further indicates that marijuana use likely has a substitutionary and complimentary relation with alcohol use (O’Hara et al., 2016), but it is more likely that marijuana is used as a complement to alcohol among 14-20 year-olds (Pape et al., 2009). Thus, risk factors for alcohol use may be closely linked to risk factors of marijuana and CAM use. Given the high rates of marijuana and CAM use in adolescents and young adults, it is important to identify risk factors, which can be then used in targeted interventions.

## The Prototype Willingness Model

One particularly suitable theoretical framework to identify risk factors for substance use among adolescents and young adults is the Prototype Willingness Model (PWM; Gerrard et. al, 2008). The PWM is a dual-process model that hypothesizes two pathways that can lead to substance use among adolescents and young adults: the reasoned pathway and the social reaction pathway. The reasoned pathway characterizes constructs that impact behavior that is planned or intended, whereas the social reaction pathway models situational influences on a behavior, for example processes during a risk-conducive situation (e.g., at a party). Cognitions in the reasoned pathway include perceived approval by others (i.e., injunctive norms), approval of behavior (i.e., attitudes) and intention or plans. Cognitions in the social reaction pathway include perceived likelihood of negative consequences from engaging in a behavior (i.e., perceived vulnerability), perceived peer’s frequency of engaging in behaviors (i.e., descriptive norms), images of peers who engage in a behavior (i.e., prototype favorability), and willingness or openness to engage in a behavior should the opportunity be presented (i.e., behavioral willingness). Of note, both pathways may operate simultaneously and although intentions and willingness are often highly correlated, they serve as independent predictors of health risk behavior (Gerrard et al., 2008; Litt et al., 2014; Todd et al., 2014). In addition, research indicates that PWM risk cognitions are often established prior to an individual engaging in a behavior (Ajzen 1985; Fishbein & Ajzen 1975; Gerrard et al., 2008), thus, cognitions related to a specific behavior may indicate a predisposition to engage in that behavior. As such, there is utility in examining PWM cognitions among substance users and non-substance users alike.

## The PWM and Substance Use

Historically, the PWM has been most commonly applied to examining alcohol use and research indicates that endorsing riskier alcohol-related cognitions has consistently been found to be significantly associated with greater alcohol use (Andrews et al., 2008; Gerrard et al., 2002; Litt & Lewis, 2016; Pomery et al., 2009; Rivis et al., 2006). While there is less literature examining PWM cognitions in relation to marijuana use, research indicates that willingness to use marijuana is prospectively related to higher frequency of use and more marijuana-related problems (Lewis et al., 2018). Greater marijuana injunctive norms (i.e., approval by friends and parents) are also prospectively related to higher frequency of marijuana use (Napper et al., 2016). Of note, one study (Linden-Carmichael et al., 2019) found that young adult SAM users reported significantly higher descriptive alcohol norms compared to alcohol-only users, which supports the importance of examining the role alcohol-related factors in relation to other substance use.

Coupled with research that indicates that alcohol is often the first substance initiated among adolescents and young adults (King & Chassin, 2007) and largely precedes marijuana and CAM use (Patrick et al., 2019), it is possible that alcohol-related PWM cognitions are associated with engagement in both marijuana and CAM use. Although there are several efficacious and evidence based prevention and early intervention efforts for alcohol use (e.g., Dimeff et al., 1999) that incorporate many constructs within the PWM, alcohol interventions to date have not been efficacious in also reducing marijuana use (White et al., 2015). However, it is possible that there are specific PWM risk cognitions related to alcohol use, that if targeted, may also reduce marijuana and CAM use. Thus, identifying key individual alcohol cognitions that could be targeted in alcohol prevention efforts that may also reduce marijuana use and CAM use is an important next step.

## Purpose

This secondary analysis of baseline data from a sample of adolescents and young adults taking part in a larger study (Lewis et al., 2020) was conducted to examine associations between alcohol, marijuana, and CAM use with 1) drinking willingness and intention, 2) cognitions in the social reaction pathway (i.e. descriptive norms, perceived vulnerability, prototype favorability and willingness), and 3) cognitions in the reasoned pathway from the PWM (i.e. injunctive norms, attitudes and intention to drink). We hypothesized that PWM alcohol cognitions in both pathways would be associated with alcohol, marijuana, and CAM use. All associations were expected to be positive with the exception of perceived vulnerability, which was expected to have a negative association to the outcomes.

## METHODS

### Participants

Participants included adolescents and young adults aged 15-25 years old (*N* = 124) who were part of a larger study examining within-person variation of drinking cognitions and alcohol use. See Lewis et al. (2020) for more information about full study design. Participants completed an online screening assessment, a verification phone call, and in-person baseline assessment and ecological momentary assessment (EMA) training session. Participants were then assessed using an EMA burst design. Data for the present analyses are from baseline assessments.

The sample was majority female (57.3%) with a mean age of 18.77 years (*SD* = 2.86). Ethnic and racial representation of the sample was 59.7% White, 15.3% Asian, 13.7% more than one race, 7.3% Black, 7.3% Hispanic/Latino, and 4.0% Other/Mixed. The majority of participants were high school or college students and 13.7% were not a current student. Those who were not a current student were 20 years or older. Of those who were current students (86.3%), 40.3% were in high school, 33.9% attended a 4-year college, 4.8% attended a 2-year college, 4.8% were attending pre-college courses in high school, 1.6% attended graduate or professional school, and 0.8% attended an alternative high school.

### Procedures

All study procedures were approved by the University’s Institutional Review Board, and no adverse events were reported. Recruitment for this study was conducted in the greater Seattle metropolitan area through online recruitment, print advertisements, participant referrals, and flyers. Interested individuals were asked to complete a brief, online screening survey to determine eligibility for the study. Participants were eligible if they were aged between 15 and 25, and reported drinking alcohol at least once a month in the past six months if 18 or older. See Lewis et al., 2020 for full eligibility criteria details). Eligible participants were then stratified based on demographic characteristics (i.e., biological sex, age, and typical number of drinks per month) to ensure a diverse sample. Within each age category (e.g., 15, 16, 17, etc.), individuals were stratified by biological sex and typical number of drinks per month (0 drinks per month, 1-5 drinks per month, 6+ drinks per month). After stratification, eligible participants completed a phone screen to verify certain information and to exclude individuals who may have provided false answers or were professional survey takers. Those with continuing eligibility following the phone screen were invited to complete an in-person training session and baseline assessment (*N* = 142). Of the 142 participants that were invited, 124 participants provided consent and completed the baseline survey and are included in the analyses. Participants earned $50 for completing the in-person training session and baseline assessment from which current data are drawn.

### Measures

#### Demographics.

Participants reported demographics, including age, biological sex (coded 0 = female and 1 = male), and student status (0 = not current student, 1 = current student).

#### Frequency of alcohol use.

Participants responded to one item (“On average, during the past 3 months, how often have you consumed alcohol?”) on a scale from 0 (never) to 11 (every day; Collins et al., 1985).

#### Frequency of marijuana use.

Participants responded to an open-ended item that asked, “During the past 3 months, on how many days did you use any kind of marijuana or hashish?” Participants entered a numerical response from 0-90 into an open-ended text-box.

#### Concurrent alcohol and marijuana use (i.e., CAM use).

Participants were asked to describe their alcohol and marijuana consumption using the following scale: 0 (I have never tried alcohol/marijuana), 1 (I have tried alcohol/marijuana, but currently do not drink/use), 2 (I am a light drinker/user), 3 (I am a moderate drinker/user), 4 (I am a heavy drinker/user).

Using these variables, CAM use was categorized into 2 (i.e., current CAM use), 1 (i.e., single use of alcohol or marijuana), and 0 (i.e., no current use of either alcohol or marijuana). The no current use group included those who tried either substance in the past, but do not currently use or never tried either substance. The single use group included those who had tried either but are currently only using either alcohol or marijuana.

#### Perceived descriptive drinking norms.

The Drinking Norms Rating Form (Baer et al., 1991) was used to assess perceived peer drinking with the question, “Consider a typical week during the last three months. How much alcohol, on average (measured in number of drinks), does a typical male/female your age drink on each day of a typical week?” Gender used in the question was same as that of the respondent. Total weekly drinks were summed for the final score.

#### Perceived vulnerability.

Perceived vulnerability was assessed with four items (adapted from Gerrard et al., 2008) used to rate perceived risk based on levels of alcohol use to the following stem: “How much do you think drinking alcohol at the varying levels (having 1 or 2 drinks nearly every day, having 1 or 2 drinks nearly every weekend, having 3 or 4 drinks each weekend, having 5 or 6 drinks each weekend) might cause you risk?” Responses were on a scale from 0 (no risk) to 3 (great risk) and the composite score was calculated (Cronbach’s alpha = 0.83).

#### Prototype favorability.

Prototype favorability was assessed by asking the degree to which six words [i.e., smart, popular (“cool”), mature, careless, attractive (good-looking), risky] describe the participant’s image of a typical heavy episodic drinker, using a scale from 0 (not at all) to 6 (extremely; Gerrard et al., 2008; Litt & Lewis, 2016). Scores for “careless” and “risky” were reverse scored, and a mean score of the six items was calculated such that higher scores reflected greater favorability (Cronbach’s alpha = 0.76).

#### Drinking willingness.

Participants were presented with a scenario that involved drinking at a party and rated their willingness to engage in five actions (adapted from Gerrard et al., 2008; Litt & Lewis, 2016). Sample items include “choose a non-alcoholic drink” and “stay and have one more drink” (Cronbach’s alpha = 0.85). Response options ranged from 0 (not at all willing) to 4 (completely willing), and mean scores were calculated for analyses.

#### Perceived injunctive drinking norms.

Participants responded to a series of 5 statements (Lewis et al., 2010) that assessed their perceptions of the typical male/female their ages (gender in question was based on same-sex of respondent) approval of drinking at various levels (e.g., trying one or two drinks, having 3 or 4 drinks each weekend). Responses were on a scale from Strongly Disapprove (1) to Strongly Approve (6). A mean of all items was computed with higher numbers indicating more peer approval of drinking (Cronbach’s alpha = 0.87).

#### Attitudes.

Participants responded to a series of six statements that assessed their approval (Lewis et al., 2010) of drinking at various levels (e.g., never drinking, trying one or two drinks, having 3 or 4 drinks each weekend). Responses were on a scale from Strongly Disapprove (1) to Strongly Approve (6). A mean of all items was computed with higher numbers indicating more approval of drinking (Cronbach’s alpha = 0.79).

#### Intentions.

Participants responded to a series of 5 statements that assessed how often they expect to drink alcohol in the next month, how many drinks they will have in one occasion and how often they expect to consume 4 or more drinks in a single occasion, and if they intend to reduce their drinking. One item was reverse scored, and the mean was calculated (Cronbach’s alpha = 0.69).

#### Data Analysis

Three models were examined for each outcome of interest (frequency of alcohol use, frequency of marijuana use, and CAM use vs. single substance use/no substance use), resulting in a total of nine models. For each of the three outcomes, the following constructs were examined in the three models: 1) willingness and intention to drink, 2) descriptive alcohol norms, perceived vulnerability, prototype favorability and willingness to drink (i.e., *social reaction pathway*, and 3) drinking attitudes, injunctive norms and intention to drink (i.e., *reasoned pathway*, respectively. Age, and sex were included as covariates in all nine models, in addition to alcohol use when examining marijuana use.

Frequency of alcohol use was treated as a continuous variable, and multiple linear regression models were used to examine the associations between alcohol use and PWM cognitions. Two participants reported missing marijuana use, and were excluded in the analysis. Preliminary analysis showed frequency of marijuana use was positively skewed (S = 2.35, K = 4.13) and over-dispersed (variance = 643.63, mean = 11.92). We observed the frequency of marijuana use had a relatively large number of zero values (39.3%), however, due to a small sample size of 122, fitting zero-inflated models (e.g., zero-inflated negative binomial model) would lead to estimation error with convergence issues. Therefore, negative binomial regression models were used to examine the effects of the PWM variables on marijuana use. In order to identify correlates of current CAM (*n* = 40) compared to single use (*n* = 46) and no current use (*n* = 35), multinomial logistic regression models were used. CAM was used as the reference group to aid in interpretation. Because there were only 3 marijuana-only users in the single-users group, these were excluded from the CAM use analysis. The VIF was below 2.03 and tolerance above 0.49 for all models and variables, indicating acceptable levels of multicollinearity (Hair et al., 2010). The ranges of values for VIF and tolerance were reported for each model in the results section.

## RESULTS

Overall, participants reported drinking an average of 3.88 ± 2.84 on a scale from 0 (never) to 11 (every day), which corresponds to two to three times per month, and using marijuana on average 12.03 ± 25.66 days during the past three months. Analyses of variance comparing age, and frequency of alcohol and marijuana use across groups showed that CAM users (M =19.58 ± 2.68) and single users (M = 20.20 ± 2.48) were older than non-users (M = 15.97 ± 0.92, *p* < 0.001; F(2,120) = 39.62, adjusted r^2^ = 0.39). CAM users did not drink more often during the past three months compared to single users (M= 4.89 ± 2.00, p = 0.05; F(2,120) = 84.86, adjusted r^2^ = 0.58). CAM users also used marijuana on more days in the past month (M = 34.4 ± 3.20) compared to single users (M = 0.98 ± 3.02). Means, standard deviations and 95% confidence intervals are shown in Table 1. Pearson correlations between all model variables are shown in Table 2.

### Frequency of Alcohol Use

All models examining frequency of alcohol use were significant (Model 1A: *F*(4,123) = 73.99, adj. r^2^ = 0.70; Model 2A: *F*(6,123) = 54.29, adj. r^2^ = 0.72; Model 3A: *F*(5,123) = 65.03, adj. r^2^ = 0.72; all *p’s* < 0.001). Parameter estimates are shown in Table 3.

#### Willingness and Intention.

Drinking intention was positively associated with frequency of alcohol use (*β* = 0.48) and age (*β* = 0.48, *p’s* < 0.001). Willingness and sex were not statistically significant (*p’s* > 0.24). VIF and tolerance values ranged from 1.07 to 1.66, and 0.60 to 0.93, respectively.

#### Social Reaction Pathway.

Older age (*β* = 0.42, *p* < 0.001) was associated with greater alcohol use. Additionally, higher descriptive norms (*β* = 0.31, *p* < 0.001), lower perceived vulnerability (*β* = −0.25, *p* < 0.001), and higher prototype favorability (*β* = 0.13, *p* = 0.03) were associated with greater alcohol use. Willingness to drink and sex were not significant (*p’s* > 0.07). VIF and tolerance values ranged from 1.19 to 1.53, and 0.64 to 0.84, respectively.

#### Reasoned Pathway.

Older age (*β* = 0.46, *p* < 0.001), positive attitude towards drinking (*β* = 0.20, *p* < 0.01) and intention to drink (β = 0.41, *p* < 0.001) were positively related to alcohol use. Injunctive norms and sex were not significantly associated with the outcome. VIF and tolerance values ranged from 1.10 to 1.88, and 0.53 to 0.91, respectively.

### Frequency of Marijuana Use

All models examining associations with frequency of marijuana use were significant (Model 1B: ***X***^2^ (5) = 171.03, Model 2B: ***X***^2^ (7) = 184.20, Model 3B: ***X***^2^ (6) = 175.66, all *p’s* < 0.001). Parameter estimates for the three models are shown in Table 4.

#### Willingness and intention.

The first model showed that frequency of drinking (incidence rate ratio IRR = 1.37, *p* < 0.001), willingness to drink (IRR = 1.19, *p* < 0.05), and drinking intention (IRR = 1.47, *p* < 0.001) were positively associated with frequency of marijuana use. Age and sex were not significantly associated with the outcome. VIF and tolerance values ranged from 1.08 to 1.67, and 0.60 to 0.93, respectively.

#### Social reaction pathway.

When including all constructs in the social reaction pathway (i.e., descriptive norms, prototype favorability, perceived vulnerability and willingness to drink), alcohol use (IRR = 1.50 , *p* < 0.001), descriptive norms (IRR = 1.08, *p* < 0.001), and willingness to drink (IRR = 1.25, *p* < 0.01) were positively associated with frequency of marijuana use. Perceived vulnerability, prototype favorability, age and sex did not show significant associations with the outcome. VIF and tolerance values ranged from 1.19 to 1.57, and 0.64 to 0.84, respectively.

#### Reasoned pathway.

When examining the constructs in the reasoned pathway, intention to drink (IRR= 1.34, *p* < 0.05) and approval of drinking (i.e., attitudes; IRR = 1.69, *p* < 0.01), in addition to alcohol use (IRR = 1.37, *p* < 0.001) were positively associated with frequency of marijuana use. Associations with injunctive norms, age and sex were not statistically significant. VIF and tolerance values ranged from 1.11 to 1.88, and 0.53 to 0.90, respectively.

### CAM Use, Single Use, and No Substance Use

All models were significant (Model 1C: ***X***^2^ (8) = 130.78, Model 2C: ***X***^2^ (12) = 117.00, Model 3C: ***X***^2^ (10) = 141.84, all *p’s* < 0.001) and explained 70-78% of total variance (Model 1C: Nagelkerke R^2^ = 0.74, Model 2C: Nagelkerke R^2^ = 0.70, Model 3C: Nagelkerke R^2^ = 0.78). The parameter estimates for the three models are shown in Table 5.

#### Willingness and intention.

The parameter estimates showed that younger participants (odds ratio; OR = 0.31, *p* < 0.01) with lower intention to drink (OR = 0.005, *p* < 0.01) were more likely to be non-users compared to CAM users. Willingness and sex were not statistically significant (*p’s* > 0.05). Single users had lower intentions to drink (OR = 0.55, *p* < 0.01) compared to CAM users. Age, sex, and willingness were not statistically significant. VIF and tolerance values ranged from 1.09 to 1.68, and 0.60 to 0.92, respectively.

#### Social reaction pathway.

Non-users were more likely to be younger (OR = 0.31, *p* < 0.01) and have lower willingness to drink (OR = 0.24, *p* < 0.01) compared to CAM users. Sex, descriptive norms, perceived vulnerability and prototype favorability were not statistically significant. Single users had lower descriptive norms (OR = 0.91, *p* < 0.05) than CAM users. Age, sex, willingness, perceived vulnerability and prototype favorability were not statistically significant. VIF and tolerance values ranged from 1.21 to 1.56, and 0.64 to 0.83, respectively.

#### Reasoned pathway.

Non-users were younger (OR = 0.37, *p* < 0.01), reported lower approval of drinking (OR = 0.15, *p* < 0.05), and lower intentions to drink (OR = 0.05, *p* < 0.001) compared to CAM users. Sex and injunctive norms were not statistically significant. Single users had lower approval of drinking (OR = 0.21, *p* < 0.01) compared to CAM users. Sex, age, injunctive norms and intentions to drink were not statistically significant. VIF and tolerance values ranged from 1.11 to 2.03, and 0.49 to 0.90, respectively.

## DISCUSSION

It is critical to identify risk factors for alcohol use, marijuana use, and CAM use among adolescents and young adults in order to address the risks associated with these behaviors (Arria et al., 2013; Hayaki et al., 2016; Suerken det al., 2016). Previous research indicates that drinking cognitions from the PWM strongly predict alcohol use outcomes (Gerrard et al., 2002; Litt & Lewis, 2016; Pomery et al., 2009; Rivis, Sheeran & Armitage, 2006). In this study, we found that several alcohol-related PWM cognitions on both social reaction and reasoned pathways were related to alcohol, marijuana, and CAM use vs. single substance use/no substance use. Intentions, descriptive norms and attitudes had significant effects on all the three outcomes, while perceived vulnerability and drinking prototype favorability were only significant for alcohol use. For all outcomes however, injunctive norms was not significantly related to frequency of use. Overall, current findings indicate that some drinking cognitions from the reasoned and social reaction pathways of PWM (i.e., attitudes towards drinking, intention and willingness to drink, and descriptive norms) may be more consistently related to marijuana and CAM use, suggesting potential common underlying cognitions regarding substance use in general.

Willingness and intention to drink were related to marijuana and CAM use, but when comparing CAM users to single users, only intention to drink was a risk factor for CAM use. This finding is consistent with previous literature summarized in a meta-analysis suggesting that intention is a stronger predictor of behavior than willingness (Todd et al., 2016). The first model in the current study indicated that drinking cognitions from both pathways are related to marijuana and CAM use. Models examining constructs from the reasoned pathway (i.e., injunctive norms, attitudes and intention) also showed that intention to drink were related to greater marijuana use frequency and that CAM users compared to single users had greater intention to drink. Taken together, results suggest adolescent and young adult substance use may be influenced by both individuals’ plan to use substance as well as situational factors such as willingness to drink and descriptive norms. Findings from this investigation also highlight that CAM users may be a unique population, with unique risk factors compared to single substance users of alcohol and marijuana, which is consistent with previous findings (Linden-Carmichael et al., 2019).

Current interventions for alcohol use and marijuana use largely focusing on addressing descriptive and injunctive norms by providing personalized normative feedback (e.g., Leeman et al., 2016; Walukevich-Dienst et al., 2019) may benefit from including feedback on intention to drink, as results indicate it may play an important role in marijuana and CAM use. Results of the present study also provide preliminary evidence that targeting specific alcohol-related risk cognitions may lead to reductions in marijuana and CAM use, a notion that should be further explored in future research.

Adolescents and young adults with more positive attitudes towards alcohol use reported greater frequency of marijuana use and were more likely to report CAM. However, perceived peer approval of drinking (i.e., injunctive norms) was not related to either frequency of marijuana use or CAM. Previous cross-sectional and longitudinal research has primarily examined the social reaction pathway (Lewis et al., 2018; Litt & Lewis, 2016) with regards to substance use, but the current investigation adds to the literature as it indicates, that at least cross-sectionally, the reasoned pathway of the PWM can also be applied to adolescent and young adult substance use. Further, results from cross-sectional (Litt & Lewis, 2016) and ecological momentary assessment studies (Lewis et al. , 2016) using the PWM highlight the importance of situational factors in adolescent and young adults substance use, and suggest that interventions may benefit from educating youth regarding strategies to resist substance use. Specifically, training on how to resist peer pressure in risk-conducive situations (e.g., parties), to reduce their overall and substance-specific willingness to use substances, and education on how to develop less risky substance use intentions in different situations may be beneficial according to the present findings.

Our findings also show that prototypes of alcohol users were not significantly related to frequency of marijuana use or CAM. This suggests that prototypes of drinkers, marijuana users, and CAM users may be different and need to be addressed differently. Some interventions targeting prototypes aim to create a healthier prototype image (Gerrard et al., 2008). The current study suggests that the kind of substance use should be considered when promoting healthier images. Similarly, other cognitions of the PWM, including perceived vulnerability, were only found to be associated with alcohol use but not marijuana or CAM use, suggesting that certain cognitions may be substance specific, whereas others may be relevant across substances.

Despite the contributions this study makes to the literature, this investigation is not without limitations. Due to the small sample size, a full simultaneous PWM model was not assessed, which is an important step for future research. Longitudinal tests of these associations would also be warranted given the mediational pathways proposed within the PWM (Gerrard et al., 2008). In addition, cognitions towards marijuana use and CAM were not assessed. Thus, we are unable to assess whether alcohol cognitions predict above and beyond other substance-specific cognitions. Because alcohol use, marijuana use, and CAM use had different measurement scales, we cannot make direct comparisons related to effect size. However, as noted, a primary aim of the current investigation was to assess whether cognitions towards one substance (in this case, alcohol) would also generalize towards other substances (in this case, marijuana and CAM) and so this particular concern is somewhat ameliorated. Furthermore, findings are based on a cross-sectional sample, and thus do not provide information on causality. In addition, the internal consistency of the intentions measure was low (Cronbach’s alpha = 0.69), which may lessen the reliability of results using that specific item.

In summary, findings from the current investigation provide further evidence that constructs from the PWM are useful in better understanding substance use in adolescents and young adults. Results highlight that specific drinking cognitions from the PWM may have utility in understanding marijuana and CAM use but that it is possible that adolescents and young adults have substance-specific cognitions. Overall, results suggest that interventions aimed at reducing adolescent and young adult substance use may be enhanced by also targeting substance-specific and cross-substance cognitions.

## Figures and Tables

**Table 1.** Age, Frequency of Alcohol and Marijuana Use among Non-Users (n = 35), Single Users (n = 46) and CAM Users (n = 40)

Age *years*			95% Confidence Interval	
	Mean	Std. Deviation	Lower	Upper
Non-Users	15.97	0.92	15.65	16.29
Single Users	20.20	2.48	19.46	20.93
CAM Users	19.58	2.68	18.72	20.43
Alcohol Use *use in past 3 months on a scale from 0 (never) to 11 (every day)*			95% Confidence Interval	
	Mean	Std. Deviation	Lower	Upper

Non-Users	0.51	0.74	0.26	0.77
Single Users	4.89	2.00	4.30	5.49
CAM Users	5.68	2.26	4.95	6.40
Marijuana Use *days in past 3 months*			95% Confidence Interval	
	Mean	Std. Deviation	Lower	Upper

Non-Users	0.35	0.98	0.01	0.70
Single Users	0.98	1.22	0.61	1.34
CAM Users	34.40	34.89	23.24	45.56

*Note.* The alcohol use scale values were labeled as follows: 0 – Never, 1 - Less than once per month, 2 - Once a month, 3 - Two times a month. 4 - Three times a month, 5 - Once a week, 6 - Twice a week, 7 - Three times a week, 8 - Four times a week, 9 - Five times a week, 10 - Six times a week, 11 - Every day.

**Table 2.** Pearson Correlations between Prototype Willingness Model Drinking Cognitions

Variable	Intentions	Willingness	Perceived Vulnerability	Descriptive Norms	Injunctive Norms	Attitudes	Prototype Favorability
Intentions		0.59**	−0.56**	0.47**	0.31**	0.63**	0.40**
Willingness			−0.48**	0.32**	0.14	0.51**	0.43**
Perceived Vulnerability				−0.40**	−0.31**	−0.59**	−0.31**
Descriptive Norms					0.50**	0.45**	0.04
Injunctive Norms						0.48**	0.18*
Attitudes							0.34**
Prototype Favorability							

Note.

* *p*<.05;

** *p*<.01.

**Table 3.** Parameter Estimates from Linear Regression Models on Frequency of Alcohol Use

Model 1A – Willingness and Intention					
*Frequency of Alcohol Use*					

				95% Confidence Interval	
Parameter	B	Std. Error	Standardized *β*	Lower	Upper
(Constant)	−8.13	0.94		−9.99	−6.27
Sex = male	−0.07	0.29	−0.01	−0.65	0.50
Age (years)	0.48	0.06	0.48***	0.36	0.60
Willingness	0.15	0.13	0.07	−0.11	0.41
Intentions	0.90	0.12	0.48***	0.66	1.14
Model 2A – Social Reaction Pathway					
*Frequency of Alcohol Use*					

				95% Confidence Interval	
Parameter	B	Std. Error	Standardized *β*	Lower	Upper

(Constant)	−5.70	1.25		−8.18	−3.23
Sex = male	−0.00	0.30	0.00	−0.59	0.59
Age (years)	0.42	0.06	0.42***	0.30	0.54
Descriptive Norms	0.10	0.02	0.31**	0.06	0.14
Perceived Vulnerability	−0.99	0.23	−0.25***	−1.45	−0.53
Prototype Favorability	0.48	0.22	0.13*	0.04	0.92
Willingness	0.22	0.12	0.11	−0.02	0.46
Model 3A – Reasoned Pathway					
*Frequency of Alcohol Use*					

				95% Confidence Interval	

Parameter	B	Std. Error	Standardized *β*	Lower	Lower

(Intercept)	−8.56	0.93		−10.40	−6.72
Sex = male	−0.19	0.05	−0.03	−0.29	−0.09
Age (years)	0.46	0.29	0.46***	−0.11	1.03
Injunctive Norms	−0.01	0.14	−0.00	−0.29	0.27
Attitudes	0.59	0.19	0.20**	0.21	0.97
Intentions	0.79	0.12	0.41***	0.55	1.03

Note.

* *p*<.05;

** *p*<.01;

*** *p*<.001.

**Table 4.** Parameter Estimates from Negative Binomial Regression on Frequency of Marijuana Use

Model 1B – Willingness and Intention			
*Frequency of Marijuana Use*			

		95% Confidence Interval for Exp(B)/IRR	
Parameter	Exp(B)/IRR	Lower	Upper
(Intercept)	0.57	0.08	4.21
Sex = male	1.15	0.73	1.81
Age (years)	0.96	0.85	1.08
Drinking Days (past 3 months)	1.37***	1.20	1.57
Willingness	1.19*	1.01	1.41
Intentions	1.47***	1.18	1.18
Model 2B – Social Reaction Pathway			
*Frequency of Marijuana Use*			

		95% Confidence Interval for Exp(B)/IRR	
Parameter	Exp(B)/IRR	Lower	Upper

(Intercept)	0.61	0.05	5.64
Sex = male	1.23	0.73	2.06
Age (years)	0.90	0.80	1.00
Drinking Days (past 3 months)	1.50***	1.31	1.71
Descriptive Norms	1.08***	1.04	1.11
Perceived Vulnerability	1.44	0.98	2.13
Prototype Favorability	1.21	0.82	1.79
Willingness	1.25**	1.06	1.48
Model 3B – Reasoned Pathway			
*Frequency of Marijuana Use*			

		95% Confidence Interval for Exp(B)/IRR	
Parameter	Exp(B)/IRR	Lower	Upper

(Intercept)	0.35	0.05	2.66
Sex = male	1.20	0.75	1.92
Age (years)	0.97	0.87	1.09
Drinking Days (past 3 months)	1.37***	1.20	1.57
Injunctive Norms	0.85	0.68	1.07
Attitudes	1.69**	1.19	2.42
Intentions	1.34*	1.06	1.70

Note.

* *p*<.05;

** *p*<.01;

*** *p*<.001.

**Table 5.** Parameter Estimates from Multinomial Logistic Regression on CAM use compared to Single Use and No Use

Model 1C – Willingness and Intention				
*CAM use compared to Single Use and No Use*				

			95% Confidence Interval for Exp(B)	
Reference group: CAM users (2)	Parameter	Exp(B)	Lower	Upper
Non-User (0)	Intercept			
	Sex = male	0.21	0.02	2.86
	Age	0.31**	0.14	0.70
	Willingness	0.27	0.07	1.01
	Intentions	0.05**	0.01	0.34
Single User (1)	Intercept			
	Sex = male	0.78	0.30	1.97
	Age	1.09	0.91	1.30
	Willingness	0.88	0.57	1.35
	Intentions	0.55**	0.35	0.86
Model 2C – Social Reaction Pathway				
*CAM use compared to Single Use and No Use*				

			95% Confidence Interval for Exp(B)	
Reference group: CAM users (2)	Parameter	Exp(B)	Lower	Upper

Non-User (0)	Intercept			
	Sex = male	1.02	0.12	8.38
	Age	0.31**	0.15	0.66
	Descriptive Norms	0.93	0.76	1.14
	Perceived Vulnerability	1.92	0.43	8.58
	Prototype Favorability	0.41	0.10	1.74
	Willingness	0.24**	0.10	0.59
Single User (1)	Intercept			
	Sex = male	0.61	0.21	1.82
	Age	1.21	0.99	1.47
	Descriptive Norms	0.91*	0.85	0.98
	Perceived Vulnerability	1.67	0.79	3.53
	Prototype Favorability	0.70	0.31	1.57
	Willingness	0.86	0.56	1.33
Model 3C – Reasoned Pathway				
*CAM use compared to Single Use and No Use*				

			95% Confidence Interval for Exp(B)	
Reference group: CAM users (2)	Parameter	Exp(B)	Lower	Upper

Non-User (0)	Intercept			
	Sex = male	0.57	0.05	6.92
	Age	0.37**	0.18	0.75
	Injunctive Norms	2.60	0.92	7.40
	Attitudes	0.15*	0.03	0.82
	Intentions	0.05***	0.01	0.24
Single Use (1)	Intercept			
	Sex = male	0.82	0.29	2.31
	Age	1.23	1.00	1.53
	Injunctive Norms	1.44	0.79	2.63
	Attitudes	0.21**	0.08	0.55
	Intentions	0.70	0.43	1.13

*Note.* The reference group was CAM users, coded as 2.

* *p*<.05;

** *p*<.01;

*** *p*<.001.
